# Effects of 12 Weeks of Resistance Training on Cardiovascular Risk Factors in School Adolescents

**DOI:** 10.3390/medicina56050220

**Published:** 2020-05-06

**Authors:** Lorrany da Rosa Santos, Silvan Silva de Araujo, Erlânyo Francisco dos Santos Vieira, Charles dos Santos Estevam, Jymmys Lopes dos Santos, Rogério Brandão Wichi, Fábio Bessa Lima, Carla Roberta Oliveira Carvalho, Felipe José Aidar, Anderson Carlos Marçal

**Affiliations:** 1 Departamento de Educação Física, Universidade Federal de Sergipe, São Cristóvão 49040-780, Brazil; lany0712@hotmail.com (L.d.R.S.); silvan.ssa@gmail.com (S.S.d.A.); erlanyo_lan@hotmail.com (E.F.d.S.V.); jymmyslopes@yahoo.com.br (J.L.d.S.); rbwichi@hotmail.com (R.B.W.); 2 Secretaria de Estado da Educação, do Esporte e da Cultura (SEED/SE), Aracaju 49075-470, Brazil; 3 Departamento de Fisiologia, Universidade Federal de Sergipe, São Cristóvão 49100-000, Brazil; cse.ufs@gmail.com; 4 Departamento de Fisiologia e Biofísica, Instituto de Ciências Biomédicas I (ICB I), Universidade de São Paulo, São Paulo 05508-000, Brazil; assebfabio@gmail.com (F.B.L.); croc@icb.usp.br (C.R.O.C.); 5 Physical Education Department and Group of Studies and Research of Performance, Sport, Health and Paralympic Sports―GEPEPS, Universidade Federal de Sergipe, São Cristóvão 49100-000, Brazil; fjaidar@gmail.com; 6 Departamento de Morfologia, Universidade Federal de Sergipe, São Cristóvão 49100-000, Brazil

**Keywords:** physical activity, physical exercise, hypertension, cardiovascular disease, resistance training

## Abstract

*Background and objectives:* The practice of physical exercise, especially resistance exercise, is important for the treatment and/or prevention of cardiovascular risk factors in adult individuals. However, there are few studies on its effects on adolescent individuals. Therefore, the aim of the present study was to evaluate the effects of applying a 12-week resistance training program on cardiovascular risk factors in adolescents. *Materials and Methods:* Thus, 122 adolescents aged 13–16 years of both genders participated in the study from school in the city of Lagarto, Sergipe (SE), Brazil, divided into two groups: Control Group (CG) and Group undergoing resistance training (RTG). Blood collection and anthropometric measurements were performed before and after the 12-week resistance training program (RTP). *Results:* After 12 weeks of the RTP in the adolescents, there was a reduction in the triglyceride variables (9.55%, *p* = 0.0286), Low-Density Lipoproteins (LDL) (5.42%, *p* = 0.0244), non-High-Density Lipoproteins (HDL) (5.40%, *p* = 0.0019), blood glucose (6.71%, *p* = 0.0040), systolic blood pressure (10.13%, *p* < 0.0001), as well as an increase in the body weight variable (1.73%, *p* = 0.0003). *Conclusions:* It was concluded that a 12-week RTP can prevent and/or alleviate the development of several chronic degenerative diseases in adulthood and that resistance training is important for maintaining the health of adolescents.

## 1. Introduction

Cardiovascular disease (CVD) is a major cause of early death in the world population [1,2,3]. Epidemiological studies have shown that about 60% to 80% of CVD occurs due to the exposure of individuals to cardiovascular risk factors (CRFs) [4]. Among them stand out the modifiable CRFs (obesity, hypertension, smoking, diabetes, sedentary lifestyle, dyslipidemia, among others), those that can be altered by an interventional process, in addition to the biological CRFs (genetic profile, sex, age), upon which we cannot perform interference [5].

These risk factors, when associated, contribute considerably to the development of heart and chronic degenerative diseases. Among the most common incidents in the population are atherosclerosis, acute myocardial infarction, coronary artery disease and sudden death, whose frequency of involvement in the population has grown considerably [5,6,7,8,9]. Its incidence generally occurs in adults aged 35 to 60 years [2,3], pointing to CVDs as a serious global public health problem [1].

Although clinical manifestations of cardiovascular disease usually occur in adulthood, recent studies state that its development originates in childhood and adolescence [6,7,10].

In children and adolescents with sedentary lifestyles, besides being susceptible to developing cardiometabolic diseases, they are prone to weight gain, hypertension, diabetes, obesity and dyslipidemia, thus contributing to the development of metabolic syndrome in these individuals [7,8,10].

Among the various forms of treatment to mitigate the incidence of cardiovascular disease, the scientific community recognizes the cardiometabolic adjustments promoted by physical exercise [8]. Among the known modalities, resistance exercise can promote beneficial effects on the body when practiced regularly [9]. Resistance exercise is characterized by systematically performing exercises with controlled volume and intensity, associated with muscle positioning, balance and control, in order to properly move the body against a resistant force [11]. It can be performed with the help of specific equipment segmented by muscle groups characteristic of the bodybuilding modality, which is widely practiced in the gym, as well as by free exercises through the use of dumbbells, elastic bands, suspension straps and mini bands [12,13].

There are reports in the literature that resistance exercise contributes to the prevention of physical inactivity and associated diseases such as dyslipidemia, hypertension, diabetes, obesity and other factors associated with the onset of heart disease [12,13,14,15]. Studies also suggest that the benefits of resistance training as a non-pharmacological and less expensive form of treatment are also capable of improving functional capacity, quality of life, improved strength level and motor coordination [13,16].

Thus, the present study aimed to verify the effects of 12 weeks of resistance training (RT) on cardiovascular risk factors in school adolescents, in order to verify the influence of RT on the reduction in CRFs in participants, in addition to recognizing the different biological risk factors of this age group.

## 2. Materials and Methods

### 2.1. Ethics Statement

Prior to conducting the research, it was necessary for the students’ parents to complete the Informed Consent Form. After this phase, all participants completed a health history questionnaire to ensure they met all inclusion criteria. All procedures performed during the research were approved by the Ethics Committee and research involving human beings of the Federal University of Sergipe, being governed by opinion number 65095317.8.0000.5546, approved in March 2017. Only after approval by the ethics committee did studies begin to be performed. In addition, the authors confirm that all ongoing and related trials for this intervention are registered on the Brazilian Clinical Trials Registry (Registro Brasileiro de Ensaios Clinicos (REBEC): http://www.ensaiosclinicos.gov.br) (accessed on 12-10-2019) and were approved in 12-02-2019 (trial number: RBR-8ZZ8FW).

### 2.2. Participants

The selected students are part of a public elementary school in the city of Lagarto in the interior of Sergipe (SE), Brazil, located in the rural area of the municipality.

The students who participated in the research were between 13 and 16 years old, totaling a number of 122 adolescents (3% of the total population), and were both (*n* = 52) male and (*n* = 70) female.

**Figure 1:**
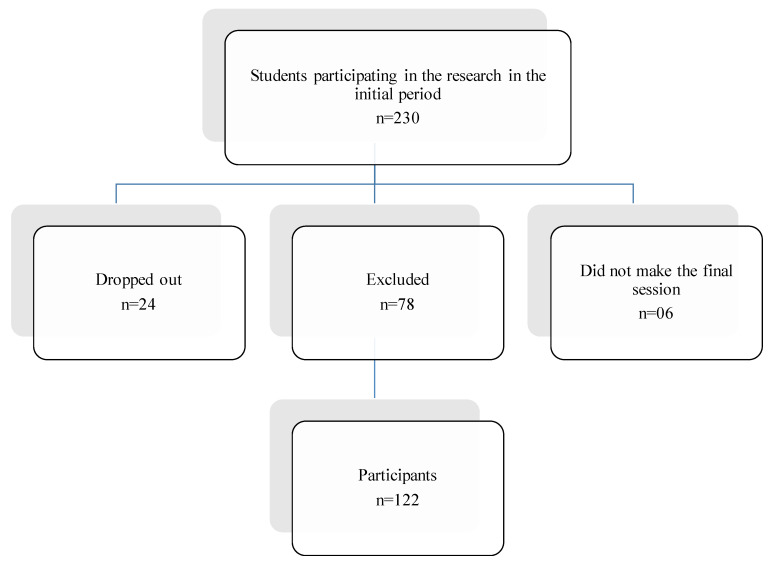
Flowchart of students who were excluded, dropped out and participated in the final survey.

Students (*n* = 122) were randomly divided into two groups: the Control Group (CG) (*n* = 61), who underwent physical education classes taught by school teachers, and the Resistance training group (RTG) (*n* = 61), who underwent a resistance training program (RTP), as shown in Figure 1.

### 2.3. Study Design

The study was conducted with adolescents from public schools aged 13 to 16 years in the city of Lagarto, SE. The age group was selected according to the classification adopted by the World Health Organization [17], which characterizes adolescence as the period between 10 and 19 years. The groups were randomly selected via a drawing carried out at the beginning of the research, this drawing was carried out by taking into account the number of female and male participants, so the groups could remain as homogeneous as possible and both groups could have an approximate number of male and female participants in their composition.

In the research development, 12 weeks of intervention with an RTP were performed (Table 1). The training program was adapted according to the model proposed by Lima [18] and designed so that the activities developed would not interfere with the maturation process of adolescents. Since many of the research participants were in the puberty age group, where hormonal variation and physical and biological development were accelerating, we could not interfere with their natural growth and development process [18].

The students evaluated were divided into two groups (CG and RTG) for the study. The CG only took the Physical Education classes prepared by the schoolteachers. The RTG group participated in a resistance training program (Table 1) for 12 weeks and were guided by two physical educators.

The interventions were always performed at the same time, referring to the Physical Education classes of each class. The interventions took place twice a week on Wednesdays and Fridays from 1 p.m. to 2 p.m./2 p.m. to 3 p.m. and 3 p.m. to 4 p.m. and were oriented by two physical educators. The other students who were allocated to the Control group took the Physical Education classes prepared by other physical educators (teachers at the schools) on the same days and times as the RTG. The activities proposed by the schoolteachers consisted of playful or recreational activities without considerable effort by the students.

Exclusion criteria were age group, presence of secondary hypertension, pregnant adolescents, adolescents with wide variation during the measurement of systolic and diastolic blood pressure values greater than or equal to 4 mmHg and adolescents who are or have been practicing resistance training over the last 6 months.

Inclusion criteria included being a registered and regular student at the aforementioned educational institution, being between 13 and 16 years old, being a student in the 6th to 9th grades of elementary school, and being willing to participate voluntarily in the research.

Therefore, the study was divided into four stages of the research:

1. The first stage: the selection of students participating in the study, separation of both groups and obtaining authorization from their parents (signing of the Informed Consent Form) to participate in the research;
2. The second stage: anamnesis through a questionnaire containing personal socioeconomic data, blood collection and anthropometric assessment (Table 1). For anthropometric analysis, we used the Pollock protocol and a suitable tape measure for body measurements of 1.5 m [19]. Body weight values were verified using an Omron HBF 514 (0–150 Kg) digital scale, height was assessed by means of a fixed Wiso E210 medical stadiometer (0–210 cm). These results were recorded in the anamnesis form of each participant, in order to serve as a basis for the calculation of the students’ Body Mass Index (BMI). Blood pressure data (SBP and DBP) were also collected by an aneroid sphygmomanometer (Ea1000 black Incoterm, Type: ACMNP-1), together with a stethoscope (Littman Classic II, 28 inches, 2114R). Both measurements were performed pre and post intervention by the same evaluators, in order to avoid any complications in the data collection. Three initial blood pressure (BP) measurements were performed in order to avoid any kind of variations that may interfere with the study, considering for the research records the average between the obtained values. Blood collection (10 mL) was performed by a competent health professional (two nursing technicians), and the student was fasting in the last 12 h prior to the collection. The samples were placed in vacuum blood collection tubes and directed to the Laboclínica Clinic Laboratory (Lagarto, SE), where the biochemical analyses were performed by a competent biochemist from the laboratory. The variables evaluated were Triglycerides (TG) Low-Density Lipoprotein (LDL) and High-Density Lipoprotein (HDL), Total Cholesterol, Non-HDL, Very Low-Density Lipoprotein (VLDL) and Blood Glucose;
3. The third stage: the induction of the RTP, which consisted of a set of 10 free exercises (Table 1) that do not exceed the maximum effort limit of each individual, so as not to impair the process of maturation, growth and development of students [12]. The exercises were performed following the order of alternation of body segments (one exercise for lower limbs, another for upper limbs and so on) [18]. The RTP carried out two days a week (Wednesday and Friday) over a period of 12 weeks [18]. The overload variable changed during the research due to the students’ physical development. In this sense, a progressive advancement of external load beyond the body weight of the individuals themselves was performed from the third week. The loads were added based on the responses obtained in the intensity evaluation performed using the Borg scale as an instrument [20], which was based on the intensity of the evaluations whose answers continued to be “Strong”. Therefore, responses with intensities lower than those previously mentioned were considered as indicative of overload. For this purpose, 2 kg, 3 kg, 4 kg and 5 kg dumbbells were used in pairs to perform the proposed exercises;
4. The fourth stage: this consisted of a post-intervention evaluation, which verified the effects and morphofunctional changes caused by the training in the students, performing a new data collection as well as previously performed Low Density Lipoprotein (LDL) and High-Density Lipoprotein (ncHDL), Total Cholesterol, Non-HDL, VLDL and Blood Glucose measurements. After the last stage of the research, the collected data will be compared in order to obtain the inferences and possible results found throughout the investigation about such a program and its influences on the reduction in adolescents’ CRFs.

### 2.4. Statistical Analysis

The sample was selected according to data obtained from the Municipal Secretariat of Education of the city of Lagarto, who reported that the municipality had about 4590 students attending the final years of elementary school at the beginning of the 2018 school year, Barbetta [21]

(1) $$n=\frac{N\times n0}{N+n0},$$

considering a sampling error of 5% of the target population, totaling a sample of 230 students (13 to 16 years old), distributed between the two larger schools in the urban area, to be evaluated during the survey. However, at the end of the research, only one school was studied, due to structural and strategic impediments. We obtained 122 participants from which the research data were evaluated, which represents, on average, 3% of the total population (Figure 1).

The data obtained from the application of the anamnesis questionnaires and registrations and the results will be expressed as means, standard deviations and confidence intervals. A previous significance level of 5% was established with a value of *p* ≤ 0.05; the results were graphically illustrated using Prism 5.0 statistical software. To ensure the privacy and integrity of students, the applied questionnaires were given codes for the analysis and reporting of results from laboratory tests.

## 3. Results

The results of this research will be presented as tables and graphs, in order to represent the values obtained in each phase of the study.

From the beginning of the activity, the RTG group increased by 3%, 14.37%, 2.33%, 10.39%, and 8.11% in the parameters age, body weight, height, body mass index (BMI), and systolic blood pressure (SBP), respectively, when compared to the CG- group (Table 2). However, the glycaemia, lipid profile of adolescents (HDL, LDL, total cholesterol, no-HDL, and VLDL), and diastolic blood pressure (DBP) were similar between CG-, and RTG- groups. The variations detected in some parameters (significant *p* value) are partly due to the biological and individual characteristics of each participant. For this reason, we chose to perform statistical analysis only within the same group (CG- versus CG+; and RTG- versus RTG+).

**Figure 2:**
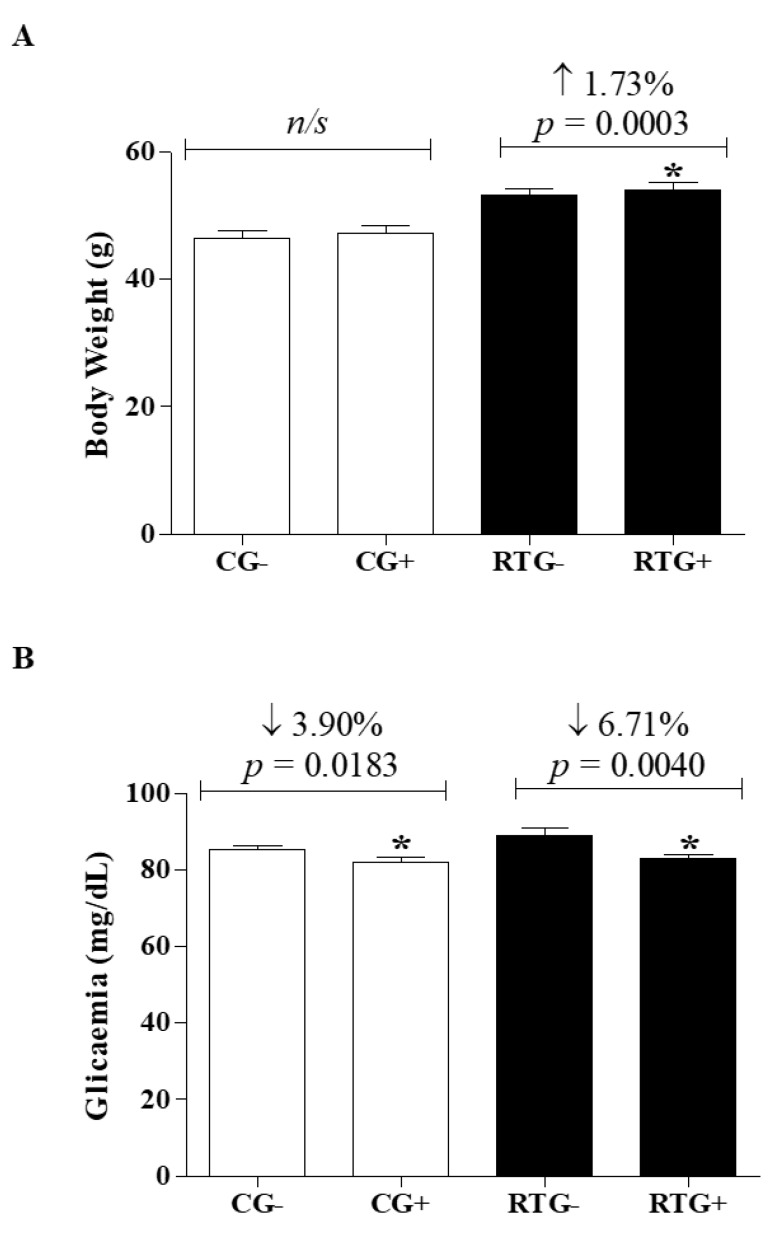
Analysis of body weight and plasma glucose of students undergoing physical activity and/or a resistance exercise training program for 12 weeks. The control group (CG) was represented by bars filled with white color where “CG-” represented day zero (beginning) and “CG+” represented the group after 12 weeks of school physical activity (*n* = 61). The group undergoing resistance training (RTG) was represented by bars filled with black color where “RTG-” represented day zero (beginning) and “RTG+” represented the group after 12 weeks of resistance training (*n* = 61). The variables body (**A**) weight and (**B**) plasma glucose were represented by the mean ± standard deviation of the mean. Student’s t test was used to evaluate the results. * = significant differences between the same group; ns = no significant difference.

Regarding body weight, the results obtained with CG after 12 weeks of physical activity (CG+ = 47.25 ± 8.855 g, *p* = 0.04544) were similar to the beginning of the activity (CG- = 46.54 ± 8.977 g) (Figure 2A). However, the group undergoing the resistance training program (RTG+ = 54.15 ± 8278 g, *p* = 0.0003) had a body weight gain of 1.73% when compared to the initial values of the same group (RTG- = 53.23 ± 8271 g) (Figure 2A).

Regarding fasting plasma glucose, there was a reduction of 3.90% and 6.71% in the control groups (CG+ = 82.05 ± 8.851 mg/dL—*p* = 0.0183) and in the group undergoing the resistance training program after the 12-week period (RTG+ = 83.03 ± 7.633 mg/dL—*p* = 0.0040) when compared with their groups at the beginning of physical activity (CG- = 85.38 ± 7.333 mg/dL) and the resistance exercise training program, respectively (RTG- = 89.00 ± 14.00 mg/dL) (Figure 2B).

**Figure 3:**
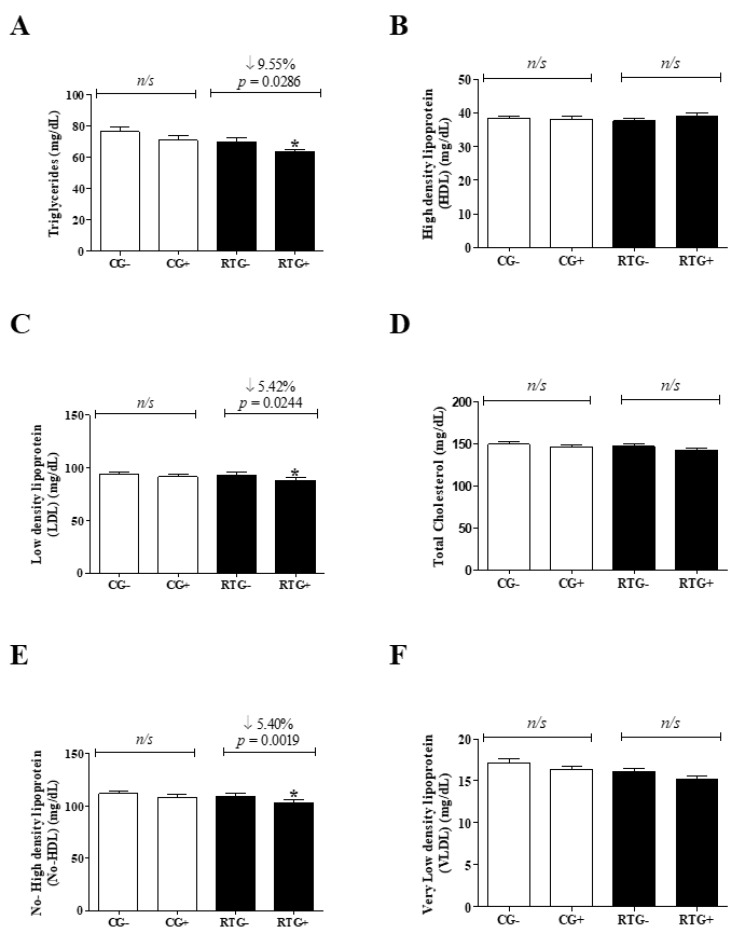
Analysis of the lipid profile of students submitted to physical activity and/or a resistance exercise training program for 12 weeks. The control group (CG) was represented by bars filled with white color where “CG-” represented day zero (beginning) and “CG+” represented the group after 12 weeks of school physical activity (*n* = 61). The group undergoing resistance training (RTG) was represented by bars filled with black color where “RTG-” represented day zero (beginning) and “RTG+” represented the group after 12 weeks of resistance training (*n* = 61). The graphs represent the mean ± standard deviation of the mean biochemical variables predicting cardiovascular risk factors: (**A**) Triglycerides, (**B**) High Density Lipoprotein (HDL), (**C**) Low Density Lipoprotein (LDL), (**D**) Total Cholesterol, (**E**) Non-HDL, (**F**) Very Low-Density Lipoprotein. Student’s t test was used to evaluate the results. * = significant differences between the same group; ns = no significant difference.

Figure 3 represents the lipid profile of adolescents. The control group, after 12 weeks of physical activity (CG +), showed similarities when compared with the same group at the beginning of physical activity (CG-) for the following variables: Triglycerides (CG- = 76.38 ± 23.27 mg/dL vs. CG+ = 70.79 ± 25.07 mg/dL, *p* = 0.1623), High Density Lipoprotein (GC- = 38.38 ± 5.352 mg/dL vs. GC+ = 38.13 ± 6.032 mg/dL, *p* = 0.7794), Low Density Lipoprotein (GC- = 93.85 ± 19.33 mg/dL vs. GC+ = 91.80 ± 18.22 mg/dL, *p* = 0.3991), cholesterol (CG- = 149.3 ± 22.50 mg/dL vs. GC+ = 146.5 ± 20.25 mg/dL, *p* = 0.3234), non-HDL (CG- = 111.9 ± 20.83 mg/dL vs. GC+ = 108.5 ± 18.89 mg/dL, *p* = 0.2121) and Very Low Density Lipoprotein (VLDL) (GC- = 17.15 ± 3.301 mg/dL vs. GC+ = 16.39 ± 3.169 mg/dL, *p* = 0.11509) (Figure 3A–F).

However, the group undergoing resistance exercise training showed a reduction of 9.55%, 5.42%, 5.40% for TG concentrations (RTG- = 69.85 ± 19.26 mg/dL vs. RTG+ = 63.18 ± 14.10 mg/dL, *p* = 0.0286), LDL (RTG- = 93.07 ± 23.59 mg/dL vs. RTG + = 88.02 ± 23.06 mg/dL, *p* = 0.0244) and no-HDL (RTG- = 109.2 ± 24.14 mg/dL vs. RTG+ = 103.3 ± 22.55 mg/dL, *p* = 0.0019) (Figure 3a,c,e, respectively). For HDL concentrations (RTG- = 37.64 ± 5.456 mg/dL vs. RTG+ = 39.18 ± 6.607 mg/dL, *p* = 0.1145), Cholesterol (RTG- = 146.9 ± 26.48 mg/dL vs. RTG + = 142.3 ± 23.17 mg/dL, *p* = 0.0524) and VLDL (RTG- = 16.15 ± 2.982 mg/dL vs. RTG+ = 15.26 ± 2.330 mg/dL, *p* = 0.0972) were similar (Figure 3b,d,f, respectively).

**Figure 4:**
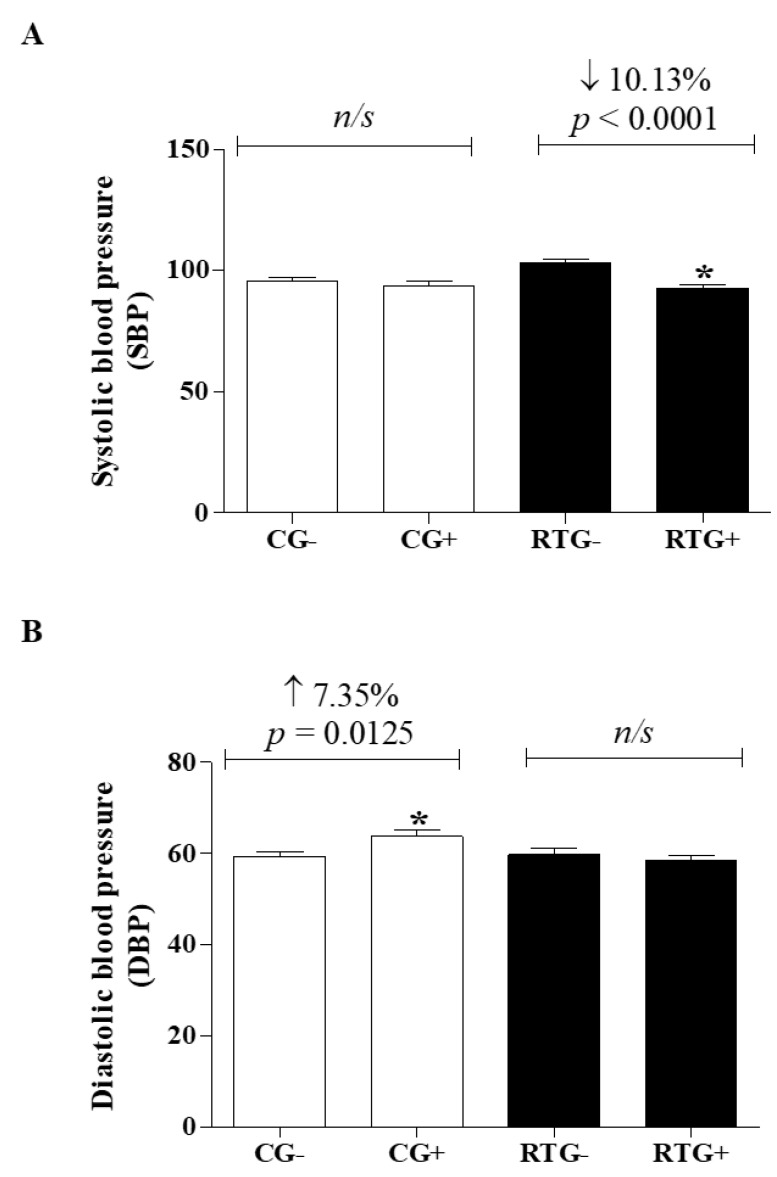
Analysis of systolic blood pressure (SBP) and diastolic blood pressure (DBP) of students undergoing physical activity and/or a resistance exercise training program for 12 weeks. The control group (CG) was represented by bars filled with white where “CG -” represented day zero (beginning) and “CG+” represented the same group after 12 weeks of school physical activity (*n* = 61). The group undergoing resistance training (RTG) was represented by bars filled with black where “RTG-” represented day zero (beginning) and “RTG+” represented the same group after 12 weeks of resistance training (*n* = 61). The graphs represent the mean ± standard deviation of the mean of the hemodynamic variables predicting cardiovascular risk factors: (**A**) Systolic Blood Pressure, (**B**) Diastolic Blood Pressure. Student’s t test was use to evaluate the results. * = significant differences between the same group; ns = no significant difference between groups.

The pressure parameters were illustrated in Figure 4. Systolic blood pressure (SBP) in the control group after 12 weeks of physical activity (CG+ = 93.68 ± 14.61 mmHg, *p* = 0.3395) was similar to the onset of activity (CG- = 95.49 ± 13.49 mmHg) (Figure 4A). However, the group undergoing resistance exercise training showed a 10.13% reduction in SBP (RTG+ = 92.75 ± 11.10 mmHg, *p* < 0.0001) (Figure 4A) when compared to the initial values of the same group (RTG- = 103.2 ± 12.52 mmHg).

Diastolic blood pressure (DBP) in the control group after 12 weeks of physical activity increased by 7.35% (CG+ = 63.69 ± 11.69 mmHg, *p* = 0.0125) when compared with the same group at the beginning of school physical activity (CG- = 59.33 ± 8372 mmHg) (Figure 4B). However, the group undergoing resistance exercise training (RTG+ = 58.59 ± 7.240 mmHg, *p* = 0.5176) was similar to the same group at the beginning of training (RTG- = 59.67 ± 11.82 mmHg).

## 4. Discussion

The parameters age, body weight, BMI, height, and SBP are statistically significant between CG- and RTG- groups. However, these results have no biological significance [22]. In addition, we must emphasize that the means of the age and BMI variables of the CG- and RTG- groups are within the normal range, as recommended by other authors [23,24]. The other parameters (glycaemia, HDL, LDL, total cholesterol, no-HDL, and VLDL, and DBP) were similar between CG-, and RTG- groups. The variations detected in some parameters are partly due to the biological and individual characteristics of each group. For this reason, we chose to perform a statistical analysis only on CG- compared to CG+, and on RTG- compared to RTG+.

Despite this, the 12-week resistance training program was effective in significantly reducing predictors of some cardiovascular risk factors in school adolescents.

Body weight in adolescents after 12 weeks of RTP intervention was increased. These results are in agreement with other authors, since this resistance training modality, when practiced regularly and continuously, is able to increase muscle mass [25,26].

The reduction in blood glucose values after 12 weeks of training may be explained by the fact that RTP is able to promote the activation of three possible mechanisms: improved insulin-independent glucose uptake [26]; improved glucose uptake by promoting positive adjustments on the signaling pathway of this hormone [27]; and/or their association (improvement of insulin dependent and independent pathways) [28,29]. Unfortunately, these hypotheses cannot be evaluated in the study groups due to the scarcity of collected samples and the fact that biopsies are difficult to obtain.

Resistance exercise training was also able to promote a reduction in the lipid profile in RTG group, attenuating triglyceride, LDL, and non-HDL concentrations when comparing the group before and after the intervention. This suggests that a training program composed of resistance exercise, when practiced in a regular and oriented manner, is able to promote adjustments in different biochemical variables in younger individuals such as adolescents [18,30]. The effectiveness of resistance exercise as a way to promote lipid profile adjustments in individuals over the age range (adults and the elderly) has been observed by several authors [18,31,32,33]. However, there are still few studies on the age range adopted in our research relating to resistance exercise and changes in these biochemical variables [26,27].

Thus, resistance exercise can and should be used as a form of prevention and/or treatment of cardiovascular diseases by favoring the reduction in cardiovascular risk factors. In particular, for the maintenance of the Triglyceride, LDL, non-HDL, Blood Glucose, SBP and DBP variables close to values recommended by regulatory organizations of the ideal health indices [23,24].

In general, for dyslipidemia, some studies suggest that the reduction in triglyceride blood concentration maintains a relationship that is inversely proportional to the increase in promoted muscle mass induced by resistance exercise training [28,29,30,31,32,33]. This does not rule out the possibility that this also occurred with adolescents who underwent our 12-week RTP.

In terms of the plasma content of circulating LDL in blood plasma, O’Donovan et al. [34] suggest that the practice of regular and continuous physical training diminishes the LDL concentration associated with a reduction in cholesterol content, which is one of the ways in which exercise exerts a protective effect concerning CRFs. Some authors suggest that RT may decrease circulating LDL-C in the bloodstream, and as a consequence, may promote reduced morbidity and mortality in the general population due to heart disease, especially in individuals who already have atherogenic dyslipidemia [11,35,36]. In our study, a significant reduction in circulating LDL values was observed in individuals after 12 weeks of resistance training intervention. Thus, we can say that the use of resistance training was able to reduce dyslipidemia and, consequently, reduce cardiovascular risks in the age group studied.

Adult subjects undergoing resistance training lasting an average of 8 to 16 weeks, as evidenced by Tibana and Prestes [37], show a reduction in Cholesterol, Triglycerides, LDL and Blood Glucose values [28,29,30]. According to these authors, these changes result from the influence of resistance training on the increase in serum LDL receptor volume, which promotes the plasma reduction of circulating LDL levels [29].

Some studies with higher frequencies and longer training periods, as proposed by Albarello et al. [32], demonstrated that HDL concentration increases when individuals engage in resistance exercise regularly [35,36]. This effect is due in part to metabolic adjustments such as decreased liver HDL degradation and increased liver synthesis [30]. However, in our study, adolescents undergoing resistance training showed no variation in HDL concentration, suggesting that an increase in the time period may induce beneficial HDL adjustments in the age group under study. It is recommended that the concentration of circulating HDL in the bloodstream should be elevated [31,32] to values above 60 mg/dl, suggesting that it can be considered as a cardioprotective agent due to its antioxidant, anti-inflammatory and platelet antiaggregant actions.

In the present study, a beneficial non-HDL adjustment was found in the group of adolescents undergoing the resistance training program when compared to the same group at the beginning of the training program. The values represented by the non-HDL variables are expressed as the sum of other biochemical variables, including LDL, VLDL and intermediate density lipoprotein (IDL). Such variables have been pointed out as strong indicators of dyslipidemia and cardiovascular risk when associated with other lipoproteins [29,30,31,32].

During resistance exercise, there is an acute elevation of blood pressure; blood pressure adjustments are regulated by the sympathetic nervous system and are mainly influenced by increases in heart rate, ejection volume and increased peripheral resistance. Consequently, after physical activity, physiological adjustments occur to reestablish the metabolic demands required during the execution of the training. Therefore, the pressure drop after training is evidenced and also relates to adjustments resulting from hemodynamic, hormonal and neural factors [38].

Blood pressure hemodynamics can be modulated by resistance training [38,39,40]. This is because RT acts favorably by increasing endothelial function, and reducing levels of systolic and diastolic pressure, heart rate, and double product values [39], which are represented by the reduction in the estimate of cardiac effort related to myocardial oxygen consumption, which RT can influence. Variations in double product values have been presented as predictors of ischemia and possibly associated with changes in ventricular functions. Resistance training, in turn, can be an important regulator of the double product at concentrations close to those desired for health maintenance.

In the present study, significant improvements in SBP values were observed in adolescents undergoing 12 weeks of resistance training. Such adjustments can be explained by the influence of RT on post-exercise hypotension (PEH) and the reduction in overall BP levels after regular intervention [40,41]. Recent studies [40,41,42] indicate the use of RT to regulate or maintain optimal blood pressure values in normotensive or hypertensive individuals. This event is due to the fact that RT in acute interventions has been shown to promote PEH. Such characteristics of this type of training point to its clinical relevance in keeping the BP of transient hypertensive individuals at lower levels during daytime intervals, when BP is usually at its highest levels [42]. Among the main adjustments promoted by resistance training are reduced peripheral vascular resistance, decreased systolic volume, reduced sympathetic activity and changes related to adrenergic endothelial sensitivity [42,43]. The limitation of our study was the reduction in the number of participants (230 to 122), due to strike affecting the students’ school, which occurred during a significant period of research development. Despite this, we advocate that maintaining BP values within the normal range from early ages, such as childhood and adolescence, is important to ensure that CRFs in later life are within the normal range recommended by modern societies.

## 5. Conclusions

It was concluded that 12 weeks of RT intervention were sufficient to present significant differences in the adolescent population evaluated in GI. Adolescents undergoing a 12-week resistance training program had beneficial adjustments in the Triglyceride, LDL, non-HDL, Blood Glucose, SBP, and DBP variables. These results suggest that RTP may be used for health maintenance and/or as an adjuvant in controlling and/or reducing CRF at an early age.

The results also demonstrate the importance of RTP in school environments. When well oriented and prepared by physical educators, RT may play an important role in avoiding possible injuries, correction and postural orientation, as performing cadence exercises at the right volume and correct intensity may provide better results. Thus, RTP, when advocated during adolescence and in a targeted manner, can prevent the development of various chronic degenerative diseases in adulthood.

## 6. Patents

We also wish to declare that we do not have any patents resulting from this work.

## Figures and Tables

**Table 1:** Resistance training program (RTP) for school adolescents (adapted from Lima [18]).

Exercises	Sets	Repetitions	Overload	Rest
Free squat	3	20	Body weight	1 min
Lunge	3	20	Body weight	1 min
Standing calf rise	3	20	Body weight	1 min
Push ups	3	20	Body weight	1 min
Bench triceps dips	3	15	Body weight	1 min
TRX pull ups	3	10	Body weight	1 min
Biceps curl (elastic band)	3	15	Body weight and band tension	1 min
Upright row (elastic band)	3	20	Body weight and band tension	1 min
Lateral raise (elastic band)	3	15	Body weight and band tension	1 min
Plank stabilization	3	30 sec ^1^	Body weight	1 min

^1^ Seconds (sec).

**Table 2:** Characterization of the adolescent groups at beginning of research (day zero).

Variables	CG-	RTG-	*p* Value
Age (years)	1300 ± 1.049	13.39 ± 0.9709 *	0.0336
Body weight (grams)	46.54 ± 8.977	53.23 ± 8.271 *	<0.0001
Height (cm)	158.3 ± 8.358	162.0 ± 6.802 *	0.0080
Body mass index (BMI) (kg/m^2^)	18.39 ± 2.765	20.30 ± 2.991 *	0.0004
Glicaemia (mg/dL)	85.38 ± 0.9389	89.00 ± 1.792	0.0759
Triglycerides (mg/dL)	76.38 ± 23.27	69.85 ± 19.26	0.0943
High density lipoprotein (HDL) (mg/dL)	38.38 ± 5.352	37.64 ± 5.456	0.4524
Low density lipoprotein (LDL) (mg/dL)	93.85 ± 19.33	93.07 ± 23.59	0.8406
Total Cholesterol (mg/dL)	149.3 ± 22.50	146.9 ± 26.48	0.5789
No-High density lipoprotein (No-HDL) (mg/dL)	111.9 ± 20.83	109.2 ± 24.14	0.5218
Very Low-density lipoprotein (VLDL) (mg/dL)	17.15 ± 3.301	16.15 ± 2.982	0.0817
Systolic blood pressure―SBP (mmHg)	95.46 ± 15.47	103.20 ± 14.31 *	0.0048
Diastolic blood pressure―DBP (mmHg)	59.33 ± 8.300	59.67 ± 7.600	0.8139

Table 2 represents the analysis of age, body weight, height, Body Mass Index (BMI), glycaemia, triglycerides, high density lipoprotein, low density lipoprotein, total cholesterol, no-high density lipoprotein, very low-density lipoprotein, systolic blood pressure (SBP), and diastolic blood pressure (DBP) of the Control Group (CG) and the Group undergoing resistance training (RTG). The “CG-” represented day zero (beginning) to the control group, and “RTG-” represented day zero (beginning) to the group undergoing resistance training. The values were represented by the mean ± standard deviation of the mean. The Student t test was use to evaluate the results. * = significant differences between the same group.
